# A Differential Role for CD248 (Endosialin) in PDGF-Mediated Skeletal Muscle Angiogenesis

**DOI:** 10.1371/journal.pone.0107146

**Published:** 2014-09-22

**Authors:** Amy J. Naylor, Helen M. McGettrick, William D. Maynard, Philippa May, Francesca Barone, Adam P. Croft, Stuart Egginton, Christopher D. Buckley

**Affiliations:** 1 Rheumatology Research Group, Centre for Translational Inflammation Research, University of Birmingham, Birmingham, West Midlands, United Kingdom; 2 Systems Science for Health, University of Birmingham, Birmingham, West Midlands, United Kingdom; 3 Faculty of Biological Sciences, University of Leeds, Leeds, West Yorkshire, United Kingdom; University of Minnesota Medical School, United States of America

## Abstract

CD248 (Endosialin) is a type 1 membrane protein involved in developmental and pathological angiogenesis through its expression on pericytes and regulation of PDGFRβ signalling. Here we explore the function of CD248 in skeletal muscle angiogenesis. Two distinct forms of capillary growth (splitting and sprouting) can be induced separately by increasing microcirculatory shear stress (chronic vasodilator treatment) or by inducing functional overload (extirpation of a synergistic muscle). We show that CD248 is present on pericytes in muscle and that CD248^-/-^ mice have a specific defect in capillary sprouting. In contrast, splitting angiogenesis is independent of CD248 expression. Endothelial cells respond to pro-sprouting angiogenic stimulus by up-regulating gene expression for HIF1α, angiopoietin 2 and its receptor TEK, PDGF-B and its receptor PDGFRβ; this response did not occur following a pro-splitting angiogenic stimulus. In wildtype mice, defective sprouting angiogenesis could be mimicked by blocking PDGFRβ signalling using the tyrosine kinase inhibitor Imatinib mesylate. We conclude that CD248 is required for PDGFRβ-dependant capillary sprouting but not splitting angiogenesis, and identify a new role for CD248 expressed on pericytes in the early stages of physiological angiogenesis during muscle remodelling.

## Introduction

Angiogenesis is the physiological process through which new blood vessels are formed from cells of the existing vasculature. This can be achieved by one of two processes, splitting or sprouting, that require different levels of pericyte involvement (reviewed in [1]–[2]). Splitting angiogenesis depends on the reorganisation of existing cell populations, where opposing vascular endothelial cells extend lamellipodia into the lumen until they contact with the opposite side of the lumen, effectively splitting the vessel in two. In contrast, sprouting angiogenesis is characterised by the migration and proliferation of endothelial cells towards an angiogenic stimulus in the tissue.

Pericytes play an essential role in stabilising the developing vessel in sprouting angiogenesis [3]. Indeed, a loss or lack of pericytes results in weaknesses in the capillaries, and has been associated with micro-aneurysms and loss of sight [4]–[5]. Pericytes are a heterogeneous population of perivascular cells located in close proximity to endothelial cells beneath a common basement membrane [2], [6]–[7]. To date, no specific pan-pericyte marker has been identified. Pericytes are defined as cells expressing either platelet-derived growth factor receptor beta (PDGFRβ), neuron glial antigen 2 (NG2), alpha smooth muscle actin (αSMA) or CD248 (endosialin) in close proximity to CD31 positive endothelial cells (reviewed in [1], [8]).

During physiological angiogenesis, platelet-derived growth factor-B (PDGF-B) secreted by endothelial cells dimerises and activates pericyte PDGFRβ, which ultimately induces their proliferation and migration towards the newly developing vessel [5]. Importantly, lack of PDGFRβ or PDGF-BB, and therefore lack of pericyte function, results in the formation of disorganised vessels leading to perinatally lethal haemorrhaging and oedema [9]–[11].

CD248 (endosialin; tumour endothelial marker 1) is a C-type lectin-like domain family member highly expressed during embryogenesis [12], and upregulated on pericytes in vascularised brain tumours and sarcomas [13]–[14]. Despite confusion in early literature on the subject (eg. [15]), CD248 expression is not seen on endothelial cells, but rather is a marker of neighbouring pericytes and stromal cells where it has the potential to influence the process of angiogenesis [16]–[17]. For example, tumour growth and large vessel formation *in vivo* are markedly reduced by genetic deletion of CD248 [18] or removal of its cytoplasmic tail [19]. Similarly, antibody blockade of CD248 interfered with pericyte migration and tube formation *in vitro* [12]. CD248 has also been shown to regulate the kinetics of vessel pruning during developmental vasculogenesis, ensuring only correctly organised and viable vessels survive [20].

Recent evidence suggests that CD248 may exert its effects through regulation of the PDGF pathway as PDGF-BB-induced phosphorylation of extracellular signal-regulated kinase (ERK), but not phosphorylation of PDGFRβ itself, was markedly diminished in CD248-deficient pericytes [21]. Thus, CD248 may function to enhance or modify PDGF-BB signalling acting downstream of PDGFRβ but upstream of ERK1/2 by an as yet unknown mechanism.

CD248 is required for pathological angiogenesis and developmental vasculogenesis [18]–[20]; however little is known about its function in physiological angiogenesis and whether this differs according to the form of capillary growth elicited (splitting *vs*. sprouting). Here we have examined the role of CD248 within skeletal muscle. Skeletal muscle displays a highly organised microvascular network with a consistent relationship between the number of capillaries and the number of muscle fibres; any increase in the capillary to fibre ratio (C:F) strongly indicates an angiogenic response [22]–[23]. We explored this relationship and demonstrated that CD248-deficient mice display a specific defect in sprouting, but not splitting, angiogenesis in skeletal muscle and demonstrate for the first time that CD248-positive pericytes are required during the early stages of physiological angiogenesis.

## Methods

### *In vivo* studies of angiogenesis

Male C57Bl/6 WT (Harlan, UK) and C57Bl/6 CD248^-/-^ mice (bred as described in Nanda *et al*. [18] donated by D.L. Huso, Johns Hopkins Medical Institutions, Baltimore, MD, USA) were studied between 8–12 weeks of age. All experiments were carried out at the University of Birmingham, UK (project licence number 40/9475) following strict guidelines governed by the UK Animal (Scientific Procedures) Act 1986 and approved by the local ethics committee (BERSC: Birmingham Ethical Review Subcommittee). Mice were housed in individually ventilated cages in groups of 3–6 individuals on a 12 hour light-dark cycle with *ad libitum* access to standard laboratory mouse chow diet and water. 6 mice were used for each experiment.

Extirpation of the tibialis anterior (TA) muscle was performed under isoflurane general anaesthesia in aseptic conditions, to produce overload of the synergist extensor digitorum longus (EDL), as previously described [24]. Alternatively, prazosin hydrochloride (50 mg/l; Tocris Bioscience, UK) supplemented with 0.5 g/l granulated sucrose (Amresco, UK) was administrated orally *ad libitum* in drinking water which was replaced every 3 days [25]. Imatinib mesylate (15 mg/ml in dH_2_0; Santa Cruz Biotechnology, USA) was administered daily by gavage, equivalent to a therapeutic dose of 150 mg/kg/day [26].

Mice were carefully monitored throughout all treatment programs by the Named Animal Care and Welfare Officer. Opiate-based pain relief was administered for 24 hours following surgical intervention. No weight loss or other adverse effects were observed in response to any of the treatments given. All treatments lasted for 7 days, after which time mice were sacrificed by cervical dislocation. The EDL was dissected immediately and either snap frozen in (i) liquid nitrogen-cooled isopentane for tissue sectioning or (ii) liquid nitrogen for gene expression analysis. All samples were stored at -80°C until use.

### Immunofluorescence

Transverse 8 µm cryosections of the EDL muscle were cut at −20°C, air dried for 1 hr, fixed in acetone at 4°C for 20 minutes and stored at −20°C until use. Sections were rehydrated and blocked at room temperature for 30 minutes in phosphate buffered saline (PBS) with 1% bovine serum albumin (PBSA; Sigma-Aldrich, UK) for capillary: fibre ratio studies, or for 15–20 minutes first in 0.05% Avidin, then 0.005% biotin and finally 10% horse serum diluted in PBS (all from Sigma) for pericyte imaging. Sections were incubated with the following primary antibodies for 1 hr at room temperature or overnight at 4°C: anti-CD31 (1∶100: AbD Serotec, BioRad, USA; clone 2H8); anti-CD248 (1∶400; clone p13, gift from Claire Isacke, The Institute of Cancer Research, London, UK); anti-NG2 (1∶75; Upstate, Merck Millipore, USA; clone 132.38); anti-αSMA (1∶100; Neomarkers, ThermoScientific, USA; clone 1A4); anti-PDGFRβ biotin (1∶100; eBioscience, USA; clone APB5), anti-collagen IV (1∶200; Abcam, Life Technologies, UK; polyclonal; ab19808), anti-phospho ERK (1∶100; Cell Signaling Technologies, UK; Phospho-p44/42 MAPK (Erk1/2) (Thr202/Tyr204) Antibody #9101). Subsequently, slides were incubated with secondary and then tertiary antibodies and Hoechst 33342 (10 µg/ml; Invitrogen, UK) for 30–60 min each before mounting with either Prolong Gold Antifade (Molecular Probes, Life Technologies, UK) or Dabco (Sigma-Aldrich, UK).

**Figure 1:**
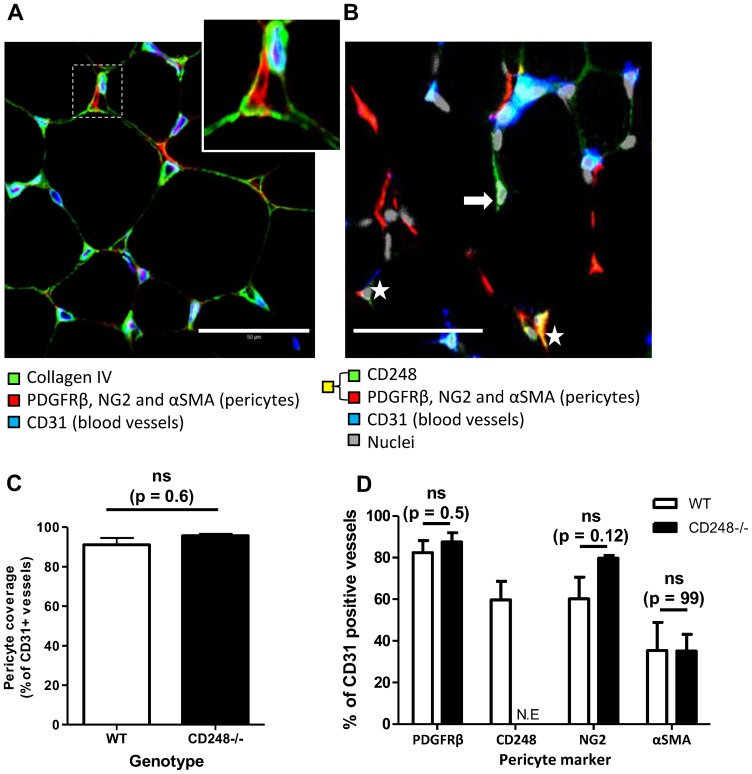
Phenotype of pericytes in wildtype and CD248 knockout skeletal muscle. Immunofluorescence and confocal microscopy of EDL muscle sections from wildtype mice stained with antibodies to either (**A**) Collagen IV (green), CD31 (blue) and pericyte markers αSMA, NG2 and PDGFRβ (all red) or (**B**) CD248 (green), CD31 (blue), pericyte markers αSMA, NG2 and PDGFRβ (all red) and nuclei (grey). CD248 was detected alone (green – marked with arrow) or co-localised with the other pericyte markers (yellow – marked with star). Enlarged region (dashed box) shows a pericyte (red) surrounded by the collagen IV basement membrane. (**C**) Pericyte coverage expressed as a percentage of CD31 positive vessels positive for ≥1 pericyte marker. (**D**) Expression of individual pericyte markers, expressed as percentage of CD31 positive vessels positive for either PDGFRβ, NG2 or αSMA. Empty bars are WT, filled bars are CD248^-/-^. Data are mean ± SEM from 3 independent animals. N.E  =  not expressed. ns = no significant difference assessed by (C) t-test or (D) ANOVA with Bonferroni post-test. Scale bars are 50 microns.

In some cases (Figure 1A, B and C), in order to visualise all pericytes at once, tissues were incubated with a cocktail of primary antibodies to PDGFRβ, NG2 and αSMA which were detected with specific secondary antibodies that all shared the same fluorophore so that all pericytes, regardless of protein expression phenotype, were visible at once. Alternatively, the pericyte markers NG2, αSMA and CD248 were analysed on separate tissue sections, each of which was counterstained with CD31 and PDGFRβ (Figure 1D).

### Fluorescence microscopy and image analysis

For quantification of capillarity, images were acquired using a Leica DM6000 fluorescence microscope controlled by Leica Advanced Fluorescence software. A fibre was defined as an isolated non-stained, convex area surrounded by collagen IV as previously described [22]. A capillary was defined as a CD31-positive structure that was <8 µm in diameter that appears either as a circle in cross section or elongated structures when sectioned obliquely. Individual branches were counted as single capillaries. Images were blinded prior to analysis. Figure S1 shows example images from each genotype for control, prazosin and extirpated mice. The differences in response to treatment become most apparent following analysis of exact capillary and fibre number, counted using Image J (NIH) and plotted as capillary to fibre ratio (C:F) as previously described [22].

To determine pericyte phenotype, confocal images were acquired for the entire muscle tissue section using a Zeiss LSM 510-UV confocal microscope, resolution (1024×768 pixels per image), magnification (x63) giving 120 µm by 120 µm for each co-localisation image. The average number of capillaries counted per field was 14 (range 3–28). Images were analysed using LSM Image Browser (Zeiss). Capillaries were defined as above, and pericytes defined as cells positive for ≥1 pericyte marker and located within 3 µm of a capillary. Pericyte coverage of capillaries was calculated as the percentage of capillaries surrounded by cell bodies or cell processes positive for ≥1 pericyte marker. For quantification of expression levels (phospho-ERK only) pixel counting was performed using Zen pro 2012 imaging software (Carl Zeiss, UK). 2 images per section were taken from 3 separate mice. Results are the sum of pixels with an expression intensity >10 normalised to untreated control tissue.

### Gene expression analysis by quantitative PCR

Messenger RNA (mRNA) was isolated from murine skeletal muscle tissue using the RNeasy Fibrous Mini Kit (following manufacturer's instructions, Qiagen, UK) and stored at -80°C until use. Isolated mRNA was converted to cDNA using High Capacity cDNA conversion kit (as per manufacturer's instructions; Applied Biosystems, UK) in a Techne TC-Plus thermal cycler (Techne, UK) and stored at −20°C until use. Gene expression was analysed by quantitative PCR (qPCR) on isolated cDNA with TaqMan 2xPCR Master Mix (Applied Biosystems, Life Technologies, UK) and FAM-labelled primers (Assay on Demand kits from Applied Biosystems, Life Technologies, UK). Samples were amplified using the 7900HT Real-Time PCR machine and analysed using SDS 2.2 (Applied Biosystems, Life Technologies, UK). Data were expressed as relative expression units (2^−ΔCt^) relative to 18S.

### Western blotting

Transverse frozen sections of EDL muscle (100×20 µm sections) were pooled for protein isolation in 50 µl RIPA buffer (Sigma Aldrich, UK) containing a protease inhibitor cocktail (Roche, UK: cOmplete, mini) phosphatase inhibitors (Roche, UK: PhosSTOP phosphatase inhibitor tablets) as per manufacturer's instructions. Samples were incubated on ice for 30 minutes with periodic vortexing. Insoluble debris was removed using QiAShredder columns (Qiagen, UK) and samples were boiled in SDS page buffer for 5 minutes. Criterion TGX precast gels (Bio-Rad, UK) were used for electrophoresis and protein was transferred using the trans-blot turbo transfer system (Bio-Rad, UK). Membranes were blocked in 5% non-fat milk in TBS containing 0.1% Tween-20. Primary antibodies were incubated at a 1∶1000 dilution performed overnight at 4°C in block. Primary antibodies were as follows: Total ERK (Cell Signaling Technologies, UK, p44/42 MAPK (Erk1/2) clone #9102), phospho-ERK (Cell Signaling Technologies, UK, Phospho-p44/42 MAPK (Erk1/2) (Thr202/Tyr204) clone #9101). Anti-rabbit HRP-linked secondary antibody (Cell Signaling Technologies, UK, #7074) was used at 1∶1000 for 1 hour at room temperature. ECL Western blotting substrate (Pierce, UK) reagent was used to visualise the resulting bands.

### Statistical analysis

Data are presented as mean ± SEM of n experiments. Variation between multiple treatments was evaluated using analysis of variance (ANOVA) followed by Bonferroni post-hoc test. Where appropriate, differences between individual treatments were evaluated by unpaired t-test unless stated otherwise in the figure legend. *P* values<0.05 were considered statistically significant.

## Results

### CD248 is expressed in skeletal muscle by a subset of pericytes

We initially characterised the phenotype of pericytes in resting skeletal muscle from wildtype (WT) and CD248^-/-^ animals. Pericytes were identified histologically with a panel of antibodies against PDGFRβ, NG2 and αSMA and clearly visible under the collagen IV basement membrane in close association with CD31-positive capillaries (Figure 1A). In both WT and CD248^-/-^ tissue almost all CD31 positive vessels within the skeletal muscle (>90%) were associated with pericyte cell bodies or processes (Figure 1C) expressing a heterogeneous mix of PDGFRβ, NG2 and αSMA (Figure 1D), thus confirming previous studies demonstrating that pericyte processes cover 99% of capillary length in normal skeletal muscle [6]. CD248 was visible on some of these pericytes (Figure 1B, examples marked with a star) and also occasionally on cells where none of the three other pericyte markers used were detectable (Figure 1B, example marked with an arrow). There were no significant differences in pericyte coverage (Figure 1C), or expression of the different pericyte markers in tissue from CD248^-/-^ compared to WT mice (Figure 1D). The majority of pericytes were positive for PDGFRβ, with 60-80% expressing NG2 and/or CD248, and ∼40% positive for αSMA (Figure 1D). Approximately 45% of CD248 positive pericytes also expressed PDGFRβ (data not shown). As expected, CD248 expression on pericytes was not expressed (NE) in CD248^-/-^ muscle tissue (Figure 1D) but this did not affect the relative expression of other markers. Following extirpation of WT mice we also found no statistically significant differences in the expression of any of the four pericyte markers (Figure S2).

### WT and CD248^-/-^ muscle display the same characteristics before treatment

**Figure 2:**
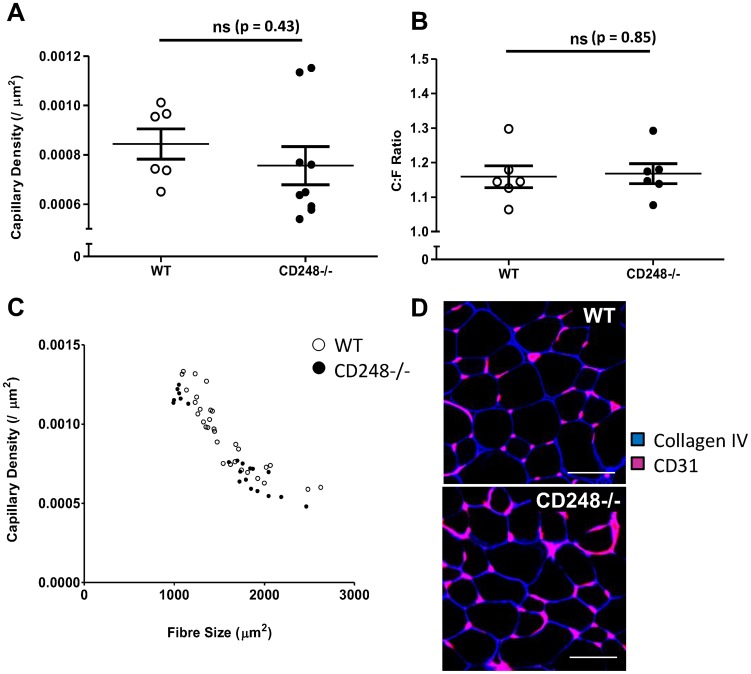
Effects of CD248^-/-^ on the architecture of skeletal muscle in control animals. (**A**) Average capillary density (capillary/µm^2^) and (**B**) capillary to fibre ratio were determined from tissue sections of untreated EDL muscle by immunofluorescence. In (**A**) and (**B**) each point represents the mean value obtained for 3–5 sections per animal. The mean for all animals is represented by the wide line and the error bars show the 95% confidence interval. (**C**) In both genotypes a non-linear relationship is seen between muscle fibre size and capillary density. Empty circles are WT, filled circles are CD248^-/-^. Each circle represents the mean value for one animal, subjected to control, extirpation or prazosin treatment. (**D**) Immunofluorescence and confocal microscopy of EDL tissue sections from untreated 8-12 week old wildtype (top) or CD248^-/-^ (bottom) mice. Sections were stained with antibodies to Collagen IV (blue) to mark the basement membrane and CD31 (red/magenta) to mark the blood vessels. Scale bars are 50 microns. ns = no significant difference by t-test.

As pericytes are critical for the stabilisation of the capillary network, we sought to examine whether genetic deletion of CD248 could affect the normal architecture of the capillary network in skeletal muscle. We observed no difference in the capillary density (CD) or the capillary to fibre ratio (C:F) in WT and CD248^-/-^ animals at baseline (Figure 2A and B respectively). Similarly, both WT and CD248^-/-^ showed the same inverse, non-linear relationship between CD and fibre size as previously described [23], with CD progressively declining as the area of the fibre increased (Figure 2C). No overt phenotypic differences in the organisation of the capillary network were observed between WT and CD248^-/-^ by confocal microscopy (Figure 2D).

### Both WT and CD248^-/-^ mice mount a splitting angiogenic response induced by prazosin treatment

**Figure 3:**
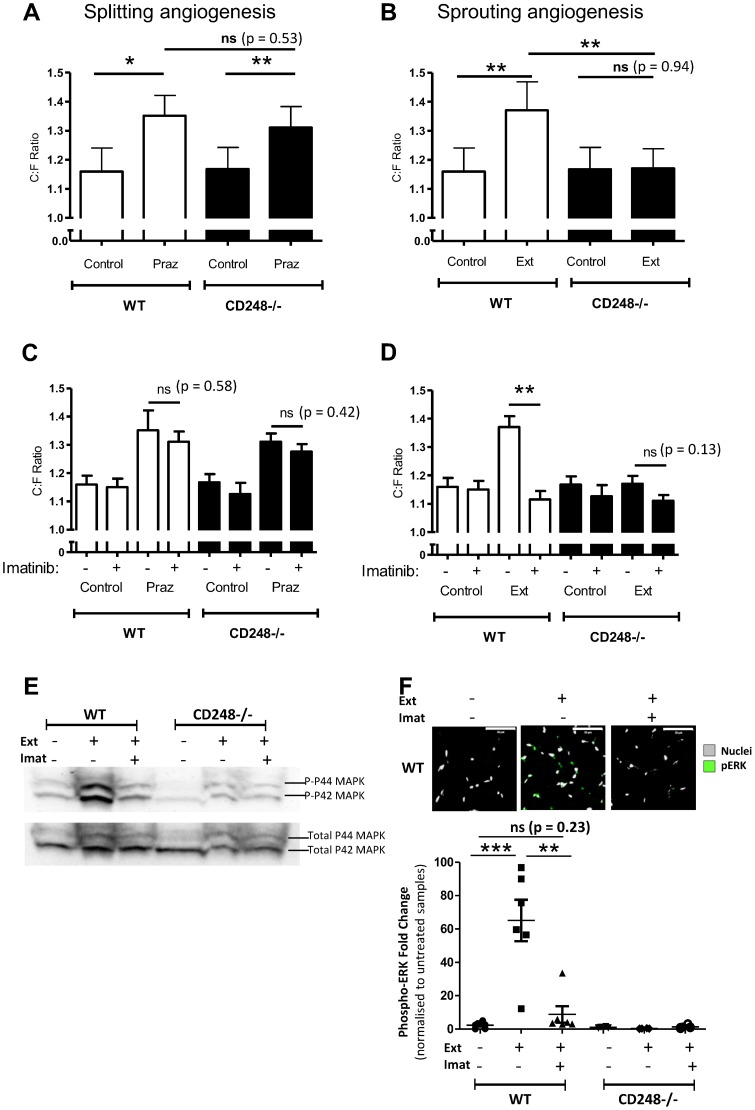
Effect of Prazosin and Extirpation treatment on muscle angiogenesis. Effects of (**A**) prazosin treatment (Pra) and (**B**) extirpation (Ext) on the capillary to fibre ratio (C:F) in wildtype and CD248^-/-^mice. The capillary to fibre ratio (C:F) was calculated for each group. Alternatively mice underwent (**C**) prazosin treatment or (**D**) extirpation surgery either alone or in the presence (+) or absence (-) of the PDGF inhibitor Imatinib (150 mg/kg/day). In each case, control animals received no treatment. Empty bars are WT, filled bars are CD248^-/-^. (**E**) Western blot of EDL muscle from WT and CD248^-/-^ mice undergoing extirpation (Ext) surgery either alone or in the presence (+) or absence (-) of the PDGF inhibitor Imatinib (Imat) at 150 mg/kg/day. Upper blot shows levels of phospho-ERK expression whilst the lower blot is a loading control showing levels of total ERK. (**F**) Top: Representative images of phospho-ERK expression by confocal microscopy from control, extirpated (Ext) and extirpated plus Imatinib (Imat). Phospho-ERK (green) nuclei (grey). Scale bar is 50 microns. Below: Pixel counts (expressed as fold change from genotype-matched control) from confocal immunofluorescence images of phosphorylated ERK from muscle sections with (+) and without (-) extirpation (Ext) and/or Imatinib (Imat) treatment. Data are mean ± SEM from 6 animals (A-D) and 3 animals (E–F). (A–D) ANOVA with Bonferroni post-test shows a significant effect of treatment and genotype on the response to stimulus. (F) Students t-test was used to identify significance *  =  *P*<0.05, **  =  *P*<0.01, ***  =  *P*<0.001, ns = non-significant.

We used prazosin to increase blood flow within skeletal muscle, thereby increasing shear stress and inducing a splitting angiogenic response [25]. As expected, we observed a significant increase in C:F ratio following treatment in WT animals and a similar increase was seen in the CD248^-/-^ muscle (Figure 3A), thus suggesting CD248 is not required for splitting angiogenesis.

### CD248^-/-^ mice are unable to mount a sprouting angiogenic response to muscle overload

Sprouting angiogenesis requires extensive pericyte involvement to co-ordinate endothelial cell migration [2], [27]–[28]. We examined the potential importance of CD248 in this process *in vivo* by using surgical extirpation of the tibialis anterior muscle. This procedure, causing overload in the EDL muscle, triggers a compensatory angiogenic response based only on sprouting that can be observed in the operated limb in rats [3], [24] and in mice [29]–[30]. Induction of sprouting angiogenesis in WT animals was demonstrated by a significant increase in C:F when compared to untreated, control mice (Figure 3B), as previously described [24]. In contrast, this response was completely ablated in CD248^-/-^ animals (Figure 3B), indicating that CD248 is required for sprouting angiogenesis in this model of muscle remodelling. Of note, we observed no gender-specific differences in these responses for either genotype (data not shown).

Recent evidence suggests that CD248 may function to enhance or modify PDGF-BB signalling by acting upstream of ERK1/2 phosphorylation in pericytes [21]. In skeletal muscle, we observed an up-regulation of ERK phosphorylation following extirpation in the WT but not in the CD248^-/-^ muscle by Western blot (Figure 3E) and immunofluorescence (Figure 3F). Interestingly, extirpation-induced ERK phosphorylation was ablated with Imatinib treatment (Figure 3E–F). Collectively these data support the concept that CD248 modulation of PDGF-BB signalling occurs upstream of ERK phosphorylation.

**Figure 4:**
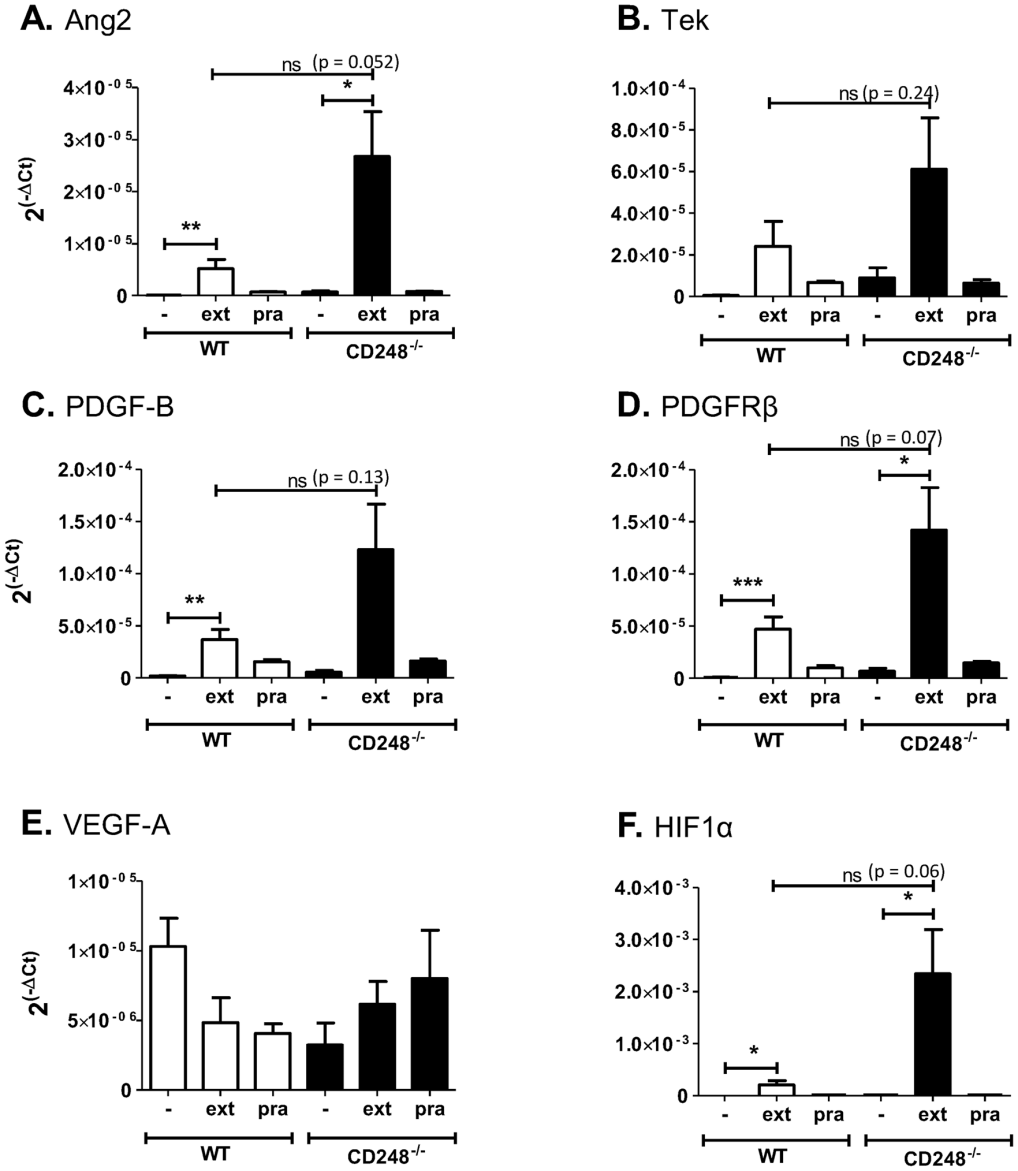
Effect of CD248 genotype on transcriptional response to Prazosin and Extirpation treatment. mRNA analysis of EDL muscle tissue by RT-PCR from WT and CD248^-/-^ mice following either no treatment control (-), extirpation (ext) or prazosin (pra) treatment. Gene transcription data were acquired for angiopoietin2 (Ang2: **A**), endothelial tyrosine kinase (TEK: **B**), platelet-derived growth factor B (PDGF-B: **C**), platelet-derived growth factor receptor β (PDGFRβ: **D**), vascular endothelial growth factor A (VEGF-A: **E**) and hypoxia-inducible factor 1α (HIF1α: **F**). Data are shown as relative expression units (2−ΔCt) relative to 18S. Data are mean ± SEM from 6 animals. ANOVA with Bonferroni post-test shows a significant effect of treatment and genotype on the response to stimulus *  =  *P*<0.05; ** =  *P*<0.01, ***  =  *P*<0.001, ns = non-significant.

We next aimed to evaluate the response of genes known to regulate the angiogenic response by using quantitative RT PCR (Figure 4). In both WT and CD248^-/-^ mice, extirpation significantly increased the levels of PDGF-B and its receptor PDGF receptor β, angiopoietin 2 and HIF1α mRNA (Figure 4). A moderate but insignificant increase was also observed for the angiopoietin receptor homologue of Tie2, TEK (Figure 4B). Prazosin treatment did not induce the expression of these genes in either WT or CD248^-/-^ animals. Tissue-wide expression of VEGF-A mRNA expression was not altered following either extirpation or prazosin treatment regardless of CD248 genotype (Figure 4E). The effects of these treatments on HIF1α, VEGF-A and Ang2 expression are broadly in agreement with previous findings [29]. To our knowledge the effect of extirpation or prazosin treatment on the expression of the other genes shown here has not been previously reported in the literature.

### Wildtype mice treated with Imatinib mesylate mirror the defect in sprouting angiogenesis observed in CD248^-/-^ mice

CD248 is known to be required for effective PDGF signalling in pericytes [21] therefore we tested whether selective inhibition of PDGF signalling could replicate the CD248^-/-^ phenotype that we observed in the sprouting angiogenic response to muscle overload. Imatinib mesylate is a tyrosine kinase inhibitor of Bcr Abl chimeric protein, c Kit and a competitive inhibitor of PDGF signalling, known to effectively block phosphorylation of the PDGF Receptor β thereby preventing downstream signalling [26]. Imatinib treatment alone had no effect on C:F ratio in untreated WT or CD248^-/-^ mice when compared to genotype-matched controls. Similarly, Imatinib treatment had no effect on the prazosin-induced increase in C:F in either WT or CD248^-/-^ mice (Figure 3C), implying that PDGF signalling is not required for splitting angiogenesis. In contrast, Imatinib completely abolished extirpation-induced angiogenesis in WT mice (Figure 3D), mirroring the effect of CD248 knockout (Figure 3B).

**Figure 5:**
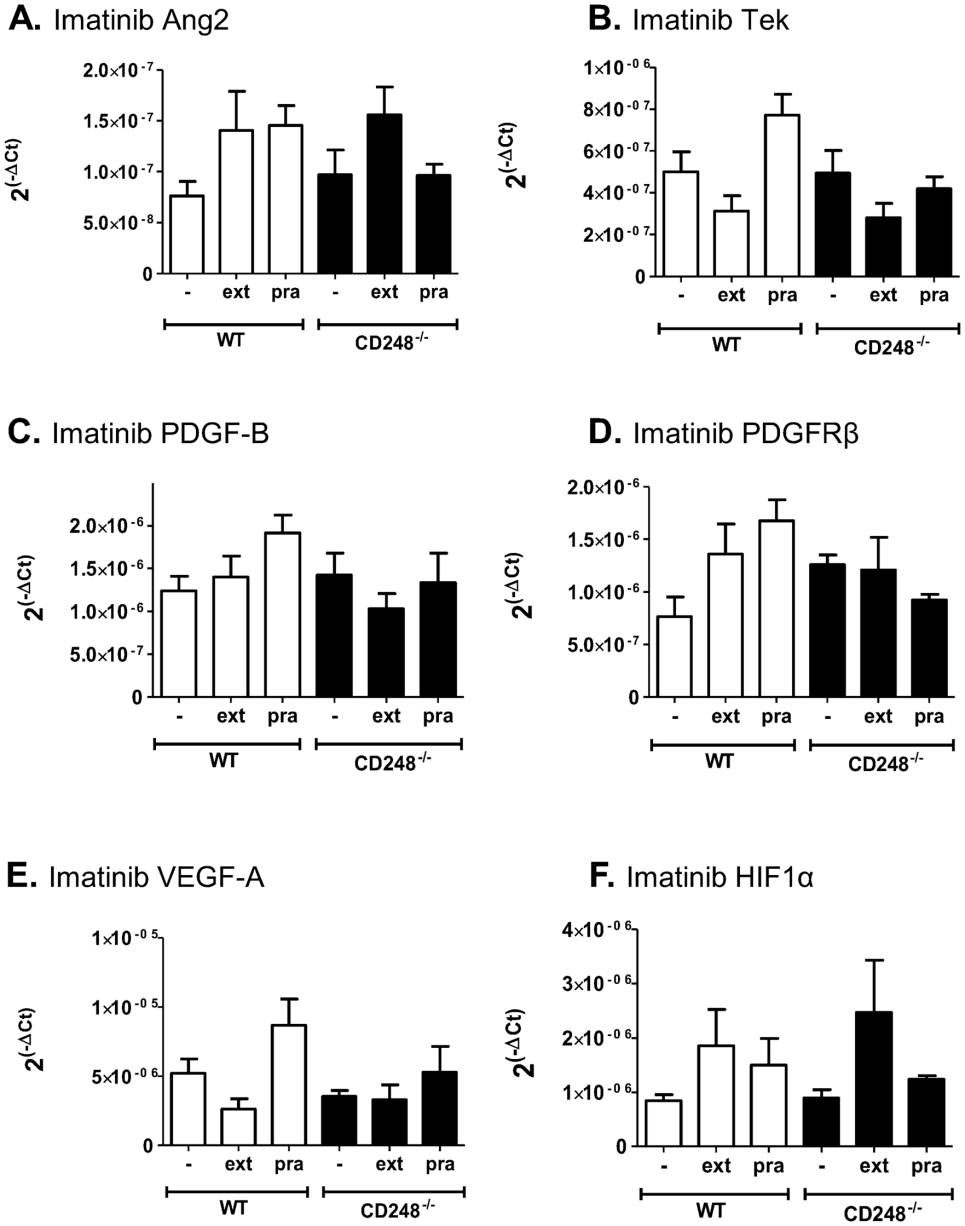
Effect of PDGF signalling inhibition by Imatinib on gene expression following Prazosin and Extirpation treatment. mRNA analysis of EDL muscle tissue by RT-PCR from WT and CD248^-/-^ mice plus no treatment control (-), extirpation (ext) or prazosin (pra) treatment. In addition, all mice were treated with Imatinib throughout the experiment. Gene transcription data were acquired for angiopoietin2 (Ang2: **A**), endothelial tyrosine kinase (TEK: **B**), platelet-derived growth factor B (PDGF-B: **C**), platelet-derived growth factor receptor β (PDGFRβ: **D**), vascular endothelial growth factor A (VEGF-A: **E**) and hypoxia-inducible factor 1α (HIF1α: **F**). Data are shown as relative expression units (2−ΔCt) relative to 18S. Data are mean ± SEM from 6 animals. ANOVA with Bonferroni post-test was performed and no significant differences were observed between any treatments or genotypes.

Interestingly, Imatinib prevented the up-regulation of PDGF-B, PDGFRβ, angiopoietin 2, TEK and HIF1α mRNA in both WT and CD248^-/-^ muscle (Figure 5), suggesting the dependency of all these genes on the PDGF signalling pathway. Again, no change was seen in VEGF-A mRNA expression following Imatinib treatment, regardless of genotype (Figure 5E).

## Discussion

Appropriate regulation of angiogenesis is crucial in allowing tissues to respond to changing physiological requirements and pericytes are known to play a crucial role in the control of these processes. In this manuscript we have shown that CD248 is expressed on some, but not all, pericytes surrounding the capillaries within resting muscle tissue and we have demonstrated a role for CD248 in sprouting angiogenesis. PDGFRβ and NG2 are widely used as pericyte markers, but are neither specific for pericytes nor expressed on all pericytes at all times [28]. While CD248 sometimes co-localised with PDGFRβ and NG2, CD31 positive capillaries were also seen closely surrounded by CD248 single-positive perivascular cells, in a location expected to be occupied by pericytes i.e. within the collagen IV basement membrane. This suggests that CD248 can be useful in identifying a pericyte subset that would otherwise be missed if only the traditional pericyte markers are used.

Angiogenesis can occur via a process of vessel splitting, where one vessel is reorganised longitudinally to become two, or by sprouting where an entirely new vessel is formed [1]. Prazosin causes an increase in microvascular flow through an α-adrenoreceptor antagonised arteriolar relaxation [31], elevating shear stress on the luminal capillary walls and triggering angiogenesis by longitudinal splitting. This is a mostly pericyte-independent process occurring through reorganisation of the endothelium with little requirement for basement membrane remodelling or an increase in pericyte number [3]. Our results demonstrate that muscle is able to effectively mount a microvascular response to increased shear stress in CD248^-/-^ mice.

CD248^-/-^ mice were unable to mount a sprouting angiogenic response to extirpation-induced overload of the EDL muscle. Extirpation causes increased stretch on myocytes, seen as an increase in sarcomere length that leads to hypertrophy and hyperplasia, releasing growth factors into the local environment, and increasing stretch on the abluminal surface of adjacent capillaries [31]. This physical stretch is the trigger for abluminal sprouting as the sole mechanism of capillary growth, a mechanotransduction process which requires pericyte migration and activation. The observation that CD248^-/-^ mice are unable to respond to these stimuli strongly suggests that CD248 expression on pericytes is required in the cascade of events leading to capillary sprout formation. Additionally, we observed that members of the PDGF, angiopoietin and HIF1α signalling pathways were upregulated only by extirpation and not by prazosin treatment. This extends the differential gene expression profile previously described between splitting and sprouting angiogenesis [29]. By Western blotting and immunofluorescence analysis we were able to show that phosphorylation of ERK occurs following extirpation in the WT but not the CD248^-/-^ muscle and that this phosphorylation can be blocked with Imatinib treatment, further implicating CD248 and the PDGF signalling cascade in this process.

**Figure 6:**
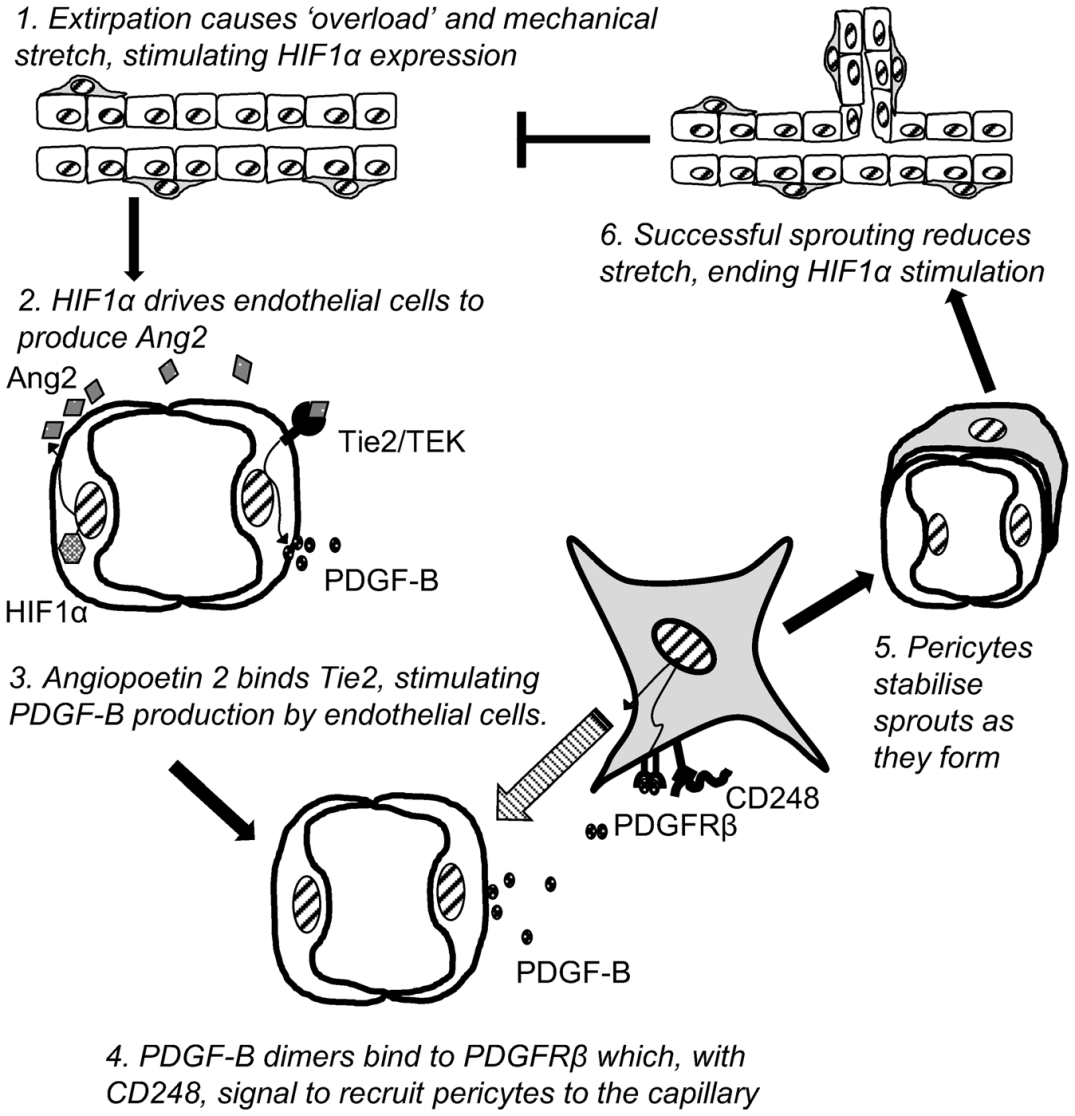
Model of the proposed role of CD248-mediated PDGF signalling in vessel sprouting. When endothelial cells are subjected to mechanical stretch, such as that seen following extirpation, the transcription factor HIF-1α is up-regulated and can bind to the HIF-binding site on the angiopoietin 2 gene. Angiopoietin 2 then binds to its receptor TEK on endothelial cells, inducing its phosphorylation. This results in upregulation of PDGF-B protein which dimerises and binds to its receptor PDGFRβ on pericytes. In the presence of CD248 this induces downstream signalling that stimulates migration of the pericyte to the endothelium and subsequent stabilisation of the capillary. When sprouting angiogenesis is successful the stretch on the capillary is reduced and HIF1α and angiopoietin gene expression can return to baseline.

Combining evidence in the literature and this most recent study, we propose the following model (Figure 6): when endothelial cells are subjected to mechanical stretch, such as that seen following extirpation, the transcription factor HIF-1α is up-regulated [32] and can bind to the angiopoietin 2 HIF-binding site resulting in up-regulation of angiopoietin mRNA [33]. Ang2 is known to be strongly upregulated at sites of active vessel remodelling [34] where it binds the receptor TEK on endothelial cells, inducing its phosphorylation [35]. This results in the up-regulation of PDGF-B protein which dimerises and binds to its receptor PDGFRβ on pericytes. In the presence of CD248, PDGFRβ signalling occurs which stimulates the migration of pericytes towards the endothelium and subsequently stabilises the capillary. Successful sprouting angiogenesis reduces the stretch experienced in the capillary bed, restoring HIF1α and angiopoietin gene expression to baseline levels. In the CD248^-/-^ mouse sprouting cannot occur due to defective PDGF signalling resulting in persistently high levels of stretch and expression of its inducible genes, HIF1α, Ang2, TEK, PDGF-B and PDGFRβ.

We have to assume that PDGF signalling can occur normally in the absence of CD248 in most situations, and in cells where CD248 is not expressed (such as endothelial cells [36]). This is evidenced by the fact that the CD248^-/-^ mouse does not phenocopy the PDGF-B or PDGFRβ knockout mice, both of which are embryonically lethal due to widespread microvascular bleeding caused by a severe shortage of vascular smooth muscle cells and pericytes [9]–[11]. In contrast, the CD248^-/-^ has no overt phenotype and no increased mortality (unpublished observations). Imatinib blocks PDGF signalling in all cell types and by measuring its effects on mRNA expression within whole muscle we were unable to distinguish CD248-mediated PDGF signalling from non-CD248-mediated. Interestingly, Imatinib blocked up-regulation of HIF1α, TEK2, Ang2, PDGF-B and PDGFRβ mRNA whilst their expression was not prevented in the CD248^-/-^ mouse. This can be explained by the non-specific nature of the inhibitor used. Recently, Chislock *et al*. [37] reported that Abelson (Abl) kinases (a target for Imatinib in addition to PDGFRβ) positively regulated TEK expression, explaining the reduction in the mRNA levels of this receptor following Imatinib treatment. In addition, Imatinib has been reported to reduce HIF1α protein expression in a model of prostate cancer by a hypoxia-independent process [38]. Given these findings, it is clear that Imatinib treatment can result in the blockade of many aspects of the HIF1α/angiopoietin/PDGF signalling process making it difficult to draw firm conclusions as to the exact role of CD248 within the cascade. Unfortunately, no specific PDGF-pathway inhibitors are available to allow us to further dissect the role of PDGF signalling in this model of sprouting angiogenesis.

Whilst the nature of the interaction of Imatinib with the PDGF pathway is understood, its influence of CD248 on this pathway remains unclear. Tomkowicz *et al*. [21] reported that CD248 interacts downstream of the PDGF receptor and upstream of ERK by an unknown mechanism and with unknown intermediaries. Our results show that the induction of PDGF-B by endothelial cells and PDGFRβ by pericytes at the gene level does not require CD248. However, downstream effects of CD248-mediated PDGF signalling that result in sprouting angiogenesis in this context seem to be dependent on CD248 expression. The intricacies of PDGF signalling have been exposed by the creation of an allelic series of PDGFRβ mice in which specific tyrosine residues were replaced with phenylalanine (reviewed in detail in [39]–[40]). All mouse strains generated were viable and fertile even when all 7 of the tyrosines that are known to be involved in PDGFRβ signalling were removed. This, compared to the PDGFRβ knockout, which is lethal at E18.5 [40] suggests that PDGFRβ has signalling capacity in the cytoplasmic tail beyond that of tyrosine phosphorylation. How CD248 interacts with the PDGF pathway has yet to be identified and is crucial in order to extend our understanding of this vital signalling pathway.

## Supporting Information

Figure S1 **Confocal images used for capillary:fibre ratio analysis.** Frozen sections from WT or CD248^-/-^ mice either untreated, extirpated or prazosin treated. Sections were stained with antibodies to collagen IV (blue) to stain basement membrane and demarcate the fibre boundaries or CD31 (red/magenta) to mark capillaries. Images are representative of 3-6 animals per group. Scale bars are 50 microns.
(TIFF)

Figure S2 **Expression of individual pericyte markers following either sham operation or extirpation, expressed as percentage of CD31 positive vessels positive for either PDGFRβ, NG2 or αSMA.** Data are mean +− SEM from 3 independent WT animals. ns = no significant difference by ANOVA with Bonferroni post-test.
(TIFF)
