# Supplementary figures and images for: A Differential Role for CD248 (Endosialin) in PDGF-Mediated Skeletal Muscle Angiogenesis
Source: PLoS One. 2014 Sep 22;9(9):e107146. doi: 10.1371/journal.pone.0107146 (PMC4171374; doi:10.1371/journal.pone.0107146)

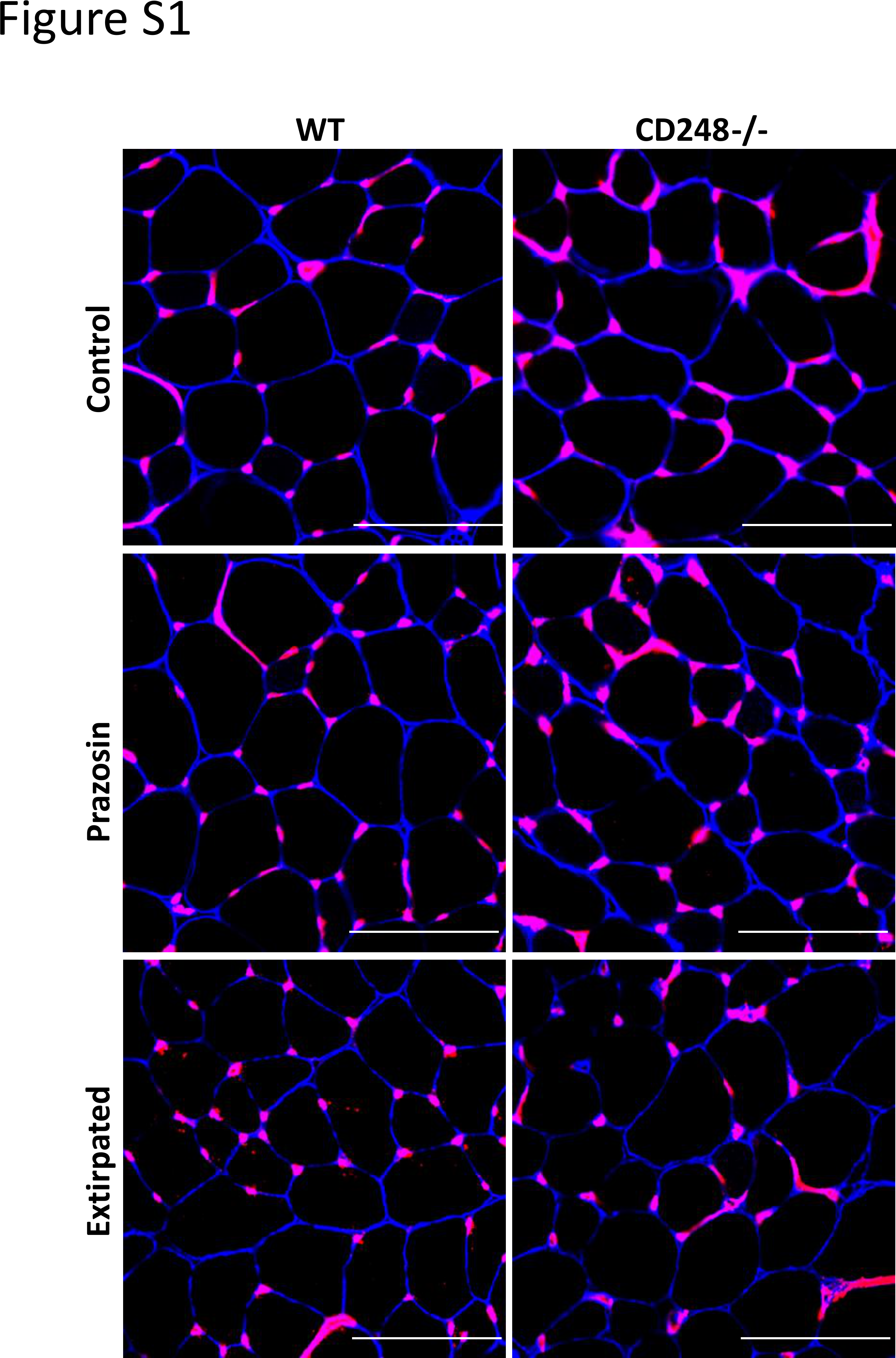

Supplement: Figure S1 — Confocal images used for capillary:fibre ratio analysis. Frozen sections from WT or CD248-/- mice either untreated, extirpated or prazosin treated. Sections were stained with antibodies to collagen IV (blue) to stain basement membrane and demarcate the fibre boundaries or CD31 (red/magenta) to mark capillaries. Images are representative of 3-6 animals per group. Scale bars are 50 microns. (TIFF) [file pone.0107146.s001.tiff]

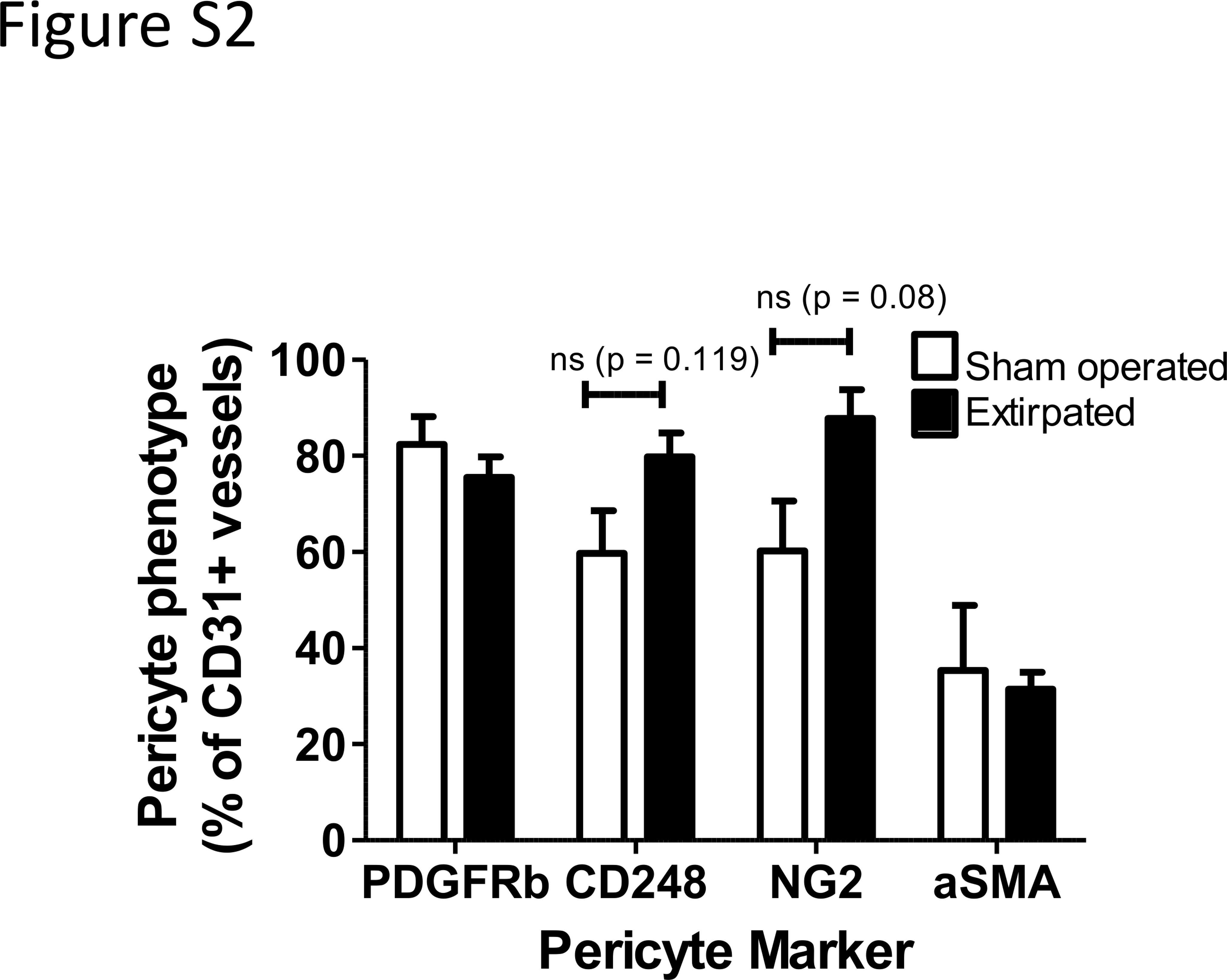

Supplement: Figure S2 — Expression of individual pericyte markers following either sham operation or extirpation, expressed as percentage of CD31 positive vessels positive for either PDGFRβ, NG2 or αSMA. Data are mean +− SEM from 3 independent WT animals. ns = no significant difference by ANOVA with Bonferroni post-test. (TIFF) [file pone.0107146.s002.tiff]
